# Combined use of susceptibility weighted magnetic resonance imaging sequences and dynamic susceptibility contrast perfusion weighted imaging to improve the accuracy of the differential diagnosis of recurrence and radionecrosis in high-grade glioma patients

**DOI:** 10.18632/oncotarget.13050

**Published:** 2016-11-03

**Authors:** Tae-Hyung Kim, Tae Jin Yun, Chul-Kee Park, Tae Min Kim, Ji-Hoon Kim, Chul-Ho Sohn, Jae Kyung Won, Sung-Hye Park, Il Han Kim, Seung Hong Choi

**Affiliations:** ^1^ Department of Radiology, Seoul National University Hospital, Seoul, Republic of Korea; ^2^ Institute of Radiation Medicine, Seoul National University Medical Research Center, Seoul, Republic of Korea; ^3^ Department of Department of Neurosurgery, Seoul National University Hospital, Seoul, Republic of Korea; ^4^ Department of Internal Medicine, Cancer Research Institute, Seoul National University Hospital, Seoul, Republic of Korea; ^5^ Department of Pathology, Seoul National University Hospital, Seoul, Republic of Korea; ^6^ Department of Radiation Oncology, Cancer Research Institute, Seoul National University Hospital, Seoul, Republic of Korea; ^7^ Center for Nanoparticle Research, Institute for Basic Science, Seoul, Republic of Korea

**Keywords:** magnetic resonance imaging, radionecrosis, recurrence, susceptibility-weighted magnetic resonance imaging sequences, dynamic susceptibility contrast perfusion-weighted imaging

## Abstract

Purpose was to assess predictive power for overall survival (OS) and diagnostic performance of combination of susceptibility-weighted MRI sequences (SWMRI) and dynamic susceptibility contrast (DSC) perfusion-weighted imaging (PWI) for differentiation of recurrence and radionecrosis in high-grade glioma (HGG). We enrolled 51 patients who underwent radiation therapy or gamma knife surgeryfollowed by resection for HGG and who developed new measurable enhancement more than six months after complete response. The lesions were confirmed as recurrence (*n* = 32) or radionecrosis (*n* = 19). The mean and each percentile value from cumulative histograms of normalized CBV (nCBV) and proportion of dark signal intensity on SWMRI (proSWMRI, %) within enhancement were compared. Multivariate regression was performed for the best differentiator. The cutoff value of best predictor from ROC analysis was evaluated. OS was determined with Kaplan-Meier method and log-rank test. Recurrence showed significantly lower proSWMRI and higher mean nCBV and 90th percentile nCBV (nCBV90) than radionecrosis. Regression analysis revealed both nCBV90 and proSWMRI were independent differentiators. Combination of nCBV90 and proSWMRI achieved 71.9% sensitivity (23/32), 100% specificity (19/19) and 82.3% accuracy (42/51) using best cut-off values (nCBV90 > 2.07 and proSWMRI≤15.76%) from ROC analysis. In subgroup analysis, radionecrosis with nCBV > 2.07 (*n* = 5) showed obvious hemorrhage (proSWMRI > 32.9%). Patients with nCBV90 > 2.07 and proSWMRI≤15.76% had significantly shorter OS. In conclusion, compared with DSC PWI alone, combination of SWMRI and DSC PWI have potential to be prognosticator for OS and lower false positive rate in differentiation of recurrence and radionecrosis in HGG who develop new measurable enhancement more than six months after complete response.

## INTRODUCTION

High-grade glioma accounts for approximately 50 % of primary malignant cerebral tumors and includes glioblastoma [World Health Organization (WHO) grade IV], anaplastic astrocytoma, mixed anaplastic oligoastrocytoma and anaplastic oligodendroglioma (WHO grade III) [1, 2]. Tumor resection followed by postoperative chemotherapy and radiation therapy (RT) is recommended as the standard of care for high-grade glioma [3]. RT has been recognized as a potent local treatment of brain tumors, but RT also damages normal brain tissue and results in radiation-related changes seen on follow-up magnetic resonance imaging (MRI) after the completion of radiation treatment [4, 5].

Several characteristic imaging features of radiation-related changes on MRI have been identified, including diffuse white matter edema-like changes, cysts and contrast-enhancing lesions [6–8]. Among these changes, newly appearing contrast-enhancing lesions, usually termed as pseudoprogression or radionecrosis, receive the attention of both clinicians and neuroradiologists because these MRI lesions can mimic the recurrence of tumors. Pseudoprogression refers to acute to subacute radiation-related changes; it typically occurs within 12 weeks and may occur up to 6 months after post-irradiation . Radionecrosis, on the other hand, encompasses late radiation-related changes occurring months to years’ post-irradiation [9]. Radionecrosis is clinically different from pseudoprogression in their late onset and possible progression with requirement for additional intervention to mitigate the effect [10]. The incidence of radionecrosis is reported between 3 % - 24 % [11].

Several studies have attempted to differentiate recurrence from radionecrosis by using dynamic susceptibility contrast (DSC) perfusion-weighted imaging (PWI). DSC PWI has been used to characterize tumor vascular physiology and hemodynamics. Tumor recurrence accompany with the formation of complex networks of abnormal blood vessels with increased permeability that appear as regions of hyper perfusion with higher blood volume. On the other hand, radionecrosis is associated with hypo perfusion because of treatment-induced vascular endothelial damage and coagulation necrosis [11]. Relative cerebral blood volume (rCBV) measurements of enhancing lesions reflect an assessment of perfusion; these measurements have been correlated with vascularity, which tends to be higher in recurrence than in radionecrosis [12, 13]. Furthermore, DSC PWI derived parameters even can be used as significant prognosticators of response in glioblastoma [14].

Susceptibility-weighted magnetic resonance imaging sequences (SWMRI) are an advanced MRI sequences which encompass susceptibility-weighted angiography (SWAN, General Electric), susceptibility weighted imaging (SWI, Siemens) and venous blood oxygen level dependent (VenoBOLD, Philips) that exploit the susceptibility differences between tissues to provide contrast in different regions of the brain, allowing for much better visualization of blood and microvessels [15]. According to preliminary reports, post-radiation changes in the brain have been related to histopathologic vascular injury or cavernous hemangioma formation [16–19]. These radiation-induced vascular alterations may result in hemorrhages, which has been reported as a fatal event in association with radiation induced temporal lobe necrosis with adjacent multiple micro hemorrhages [20].We therefore assumed that SWMRI could thus provide additional information to differentiate recurrence from radionecrosis.

In the present study, we assessed the predictive power for overall survival (OS) and the diagnostic performance of the combined use of SWMRI and DSC PWI for the differential diagnosis of recurrence from radionecrosis in high-grade glioma patients who were treated with near-total tumor removal followed by RT and who developed new enhancing lesions six months or more after a complete response.

## RESULTS

**Figure 1:**
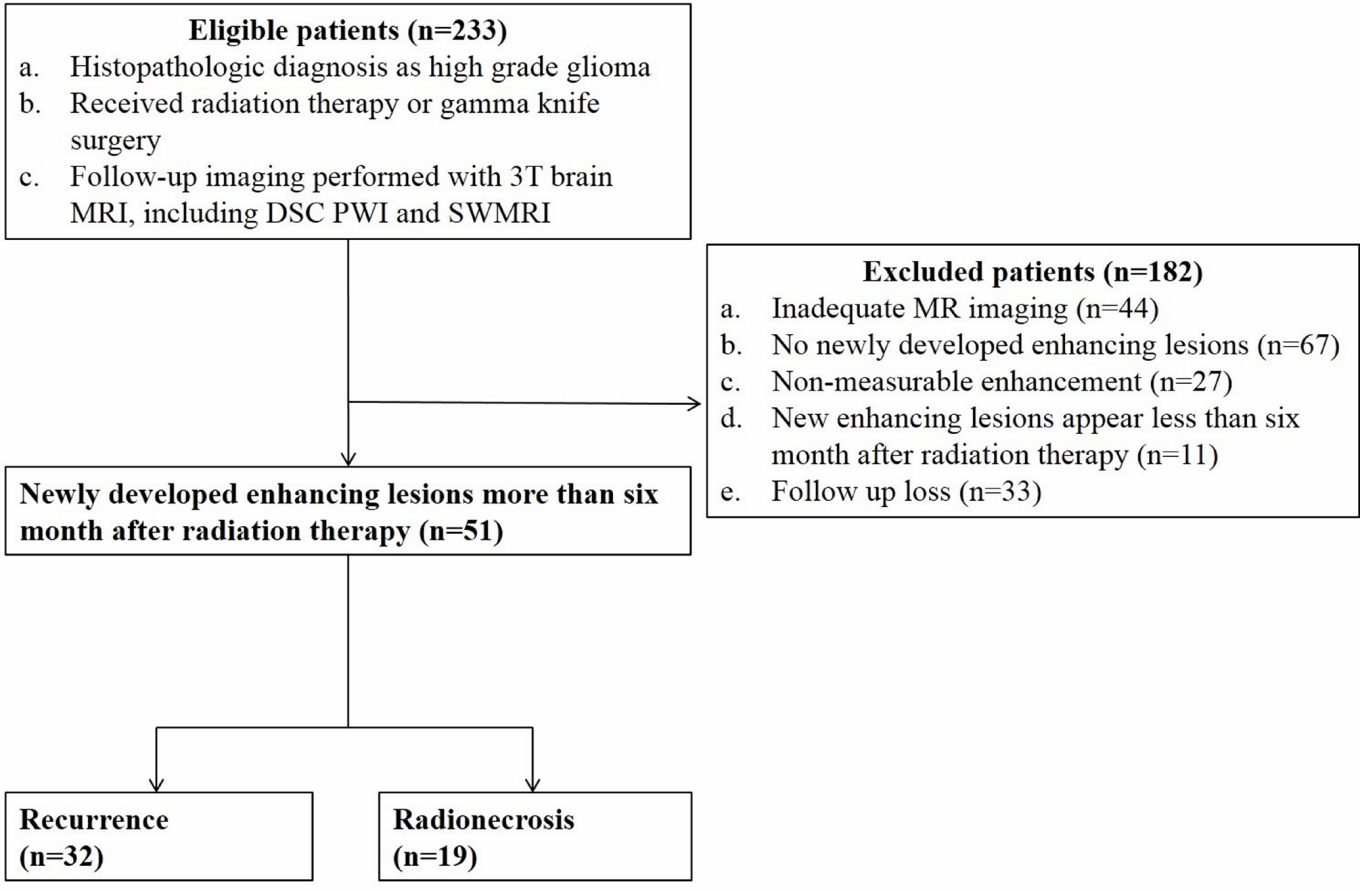
Flow diagram of patient selection with inclusion and exclusion criteria Note: DSC PWI = dynamic susceptibility contrast perfusion-weighted imaging, SWMRI = susceptibility-weighted magnetic resonance imaging sequences

Among 51 patients enrolled in the study, 32 with recurrence (histologic confirmation: 12, radiologic conclusion: 20), and 19 patients with radionecrosis (histologic confirmation: 7, radiologic conclusion: 12) were verified (Figure 1). Between the two groups, the distribution of glioblastoma (WHO grade IV) and other high-grade gliomas (WHO grade III) showed a significantly different distribution, and a higher incidence of glioblastoma was observed in the recurrence. None of the clinical parameters including age, radiation dose, time after the completion of RT, MGMT promoter methylation status, and Karnofsky performance score when follow-up MRI was taken differed between the two groups. The summarized data are shown in Table 1.

**Table 1:** Clinical characteristics of patients

	Total	Recurrence	Radionecrosis	*P* value
**Total number of patients**	n = 51	n = 32	n = 19	
** Glioblastoma**		22	7	***0.040***^†§^
** Anaplastic astrocytoma**		9	6	
** Anaplastic oligocytoma**		1	2	
** Anaplastic oligoastrocytoma**		0	4	
**Age (years)**	52.9 (±11.6)	52.6 (±13.1)	53.4 (±8.9)	0.816*
**Sex**				0.246^†^
** Male**	30	21	9	
** Female**	21	11	10	
**Confirmation**				1.000^†^
** Histologic**	19	12	7	
** Radiologic**	32	20	12	
**Radiation dose (Gy)**	59.3 (±4.7)	59.1 (±4.0)	59.8 (±5.8)	0.583*
**Time after RT (days)**	704.2 (±671.7)	583.5 (±364.6)	907.5 (±977.2)	0.096*
**Karnofsky performance score**	81.3 (±15.4)	82.0 (±15.0)	80.0 (±16.6)	0.647*
**MGMT promoter methylation (%)**	38 (74.5 %)	21 (65.6 %)	17 (89.4 %)	0.096^†^

Note: Unless otherwise indicated, data in parentheses are 95 % confidence intervals; Abbreviations: RT = radiation therapy, MGMT = methylguanine methyltransferase

* The difference between the two groups was evaluated by using Student's unpaired t-test.

^†^ The difference between the two groups was evaluated by using the Fisher's exact test.

^§^ Glioblastoma (WHO grade IV) and other high-grade gliomas (WHO grade III) were compared.

Among the calculated 70th, 90th, 95th and 99th values of the cumulative nCBV histogram, the nCBV90 (the 90th percentile) exhibited the highest AUC (0.863, *P* = 0.001) for differentiating recurrence from radionecrosis. Recurrence showed a significantly higher mean nCBV and nCBV90 than radionecrosis (3.42 *vs*. 1.18, mean nCBV, *P* = 0.002; 5.98 *vs*. 1.97, nCBV90, *P* = 0.001, respectively). Regarding proSWMRI, radionecrosis had a significantly higher mean proSWMRI (%) (42.67 *vs*. 9.3, *P* < 0.001) (Table 2).

**Table 2:** Quantitative Parameters and ROC analysis in Recurrence and Radionecrosis

	Recurrence (n = 32)	Radionecrosis (n = 19)	*P* value*	AUC	Cutoff Value	Sensitivity (%)^†^	Specificity (%)^†^	Accuracy (%)^†^
**Mean nCBV**	3.42 (±2.89)	1.18 (±0.83)	***0.002***	0.859 (0.732, 0.940)	1.07	90.6 (29/32)	68.4 (13/19)	82.3 (42/51)
**nCBV90**	5.98 (±4.86)	1.97 (±1.28)	***0.001***	0.863^⊙^ (0.738, 0.943)	2.07	87.5 (28/32)	73.7^¥^ (14/19)	82.3 (42/51)
**proSWMRI (%)**	9.3 (±9.79)	42.67 (±24.64)	***< 0.001***	0.900 (0.783, 0.966)	15.76	75.0 (24/32)	89.5 (17/19)	80.3 (41/51)
**nCBV90 + proSWMRI**				0.939^⊙^ (0.835, 0.987)		71.9 (23/32)	100 (19/19)	82.3 (42/51)

Note: Unless otherwise indicated, data in parentheses are 95 % confidence intervals; Abbreviations: nCBV = normalized relative cerebral blood volume, nCBV90 = the 90^th^ percentile values of the cumulative nCBV histogram, proSWMRI = proportion of dark signal intensity on susceptibility-weighted magnetic resonance imaging sequences, ROC = receiver operating curve, AUC = area under ROC.

*The *P* value for the mean comparison of each parameter between two groups. The difference between the two groups was evaluated using Student's unpaired t-test.

† Sensitivity, specificity and accuracy for identification of recurrence. Numbers in parentheses are raw data.

⊙ The AUC comparison did not reach statistical significance (*p* = 0.175).

^¥^The specificity between nCBV90 and nCBV90+proSWMRI showed significant difference by the Fisher's exact test (*p* = 0.046).

The interclass correlation coefficients for mean nCBV, and nCBV90 and proSWMRI were 0.775 (95 % confidence interval [CI]: 0.56, 0.88), 0.857 (95 % confidence interval [CI]: 0.72, 0.92) and 0.943 (95 % confidence interval [CI]: 0.89, 0.97), respectively. The coefficients of variation of quantitative agreement for these parameters were 16.9 %, 16.6 % and 33.3 %, respectively.

ROC curve analysis of the mean nCBV, nCBV90 and proSWMRI was performed for the differentiation, respectively. The mean nCBV cutoff value of 1.07 exhibited sensitivity, specificity and accuracy values of 90.6 % (29 of 32 patients with recurrence), 68.4 % (13 of 19 patients with radionecrosis), and 82.3 % (42 of all 51 patients), respectively. The nCBV90 cutoff value of 2.07 showed the same accuracy (82.3 %) but different sensitivity (87.5 %, 28 of 32 patients with recurrence) and specificity (73.7 %, 14 of 19 patients with radionecrosis). The proSWMRI cutoff value of 15.76 % showed the sensitivity, specificity and accuracy values of 75.0 % (24 of 32 patients with recurrence), 89.5 % (17 of 19 patients with radionecrosis), and 80.3 % (41 of all 51 patients), respectively (Table 2).

The multivariate logistic regression analysis using tumor grade (III *vs*. IV), mean nCBV, nCBV90 and proSWMRI revealed that nCBV90 and proSWMRI were two independent variables for the differentiation between recurrence and radionecrosis.

To evaluate the diagnostic performance of the combination of nCBV90 and proSWMRI, ROC curve comparison between nCBV90 alone and the combination of nCBV90 and proSWMRI was performed. AUC was larger when nCBV90 and proSWMRI were combined than for nCBV90 alone but without statistical significance (0.939 *vs*. 0.863, *P* = 0.175). The combination of nCBV90 and proSWMRI achieved 71.9 % sensitivity (23/32), 100.0 % specificity (19/19) and 82.3 % accuracy (42/51) using the best cut-off values (nCBV90 of 2.07 and proSWMRI of 15.76 %) from the ROC analysis (Table 2). The specificity of the combination of nCBV90 and proSWMRI was significantly higher than that of nCBV90 alone (100 % (19/19) *vs*. 73.7 % (14/19)) (*P* = 0.046). With the LOOCV test, the accuracy of the combination of nCBV90 and proSWMRI in predicting recurrences and radionecrosis was 80.3 % (41/51).

In the subgroup analysis, all the cases of radionecrosis (*n* = 5) with nCBV90 > 2.07 showed obvious hemorrhage (proSWMRI > 32.9 %).

**Figure 2:**
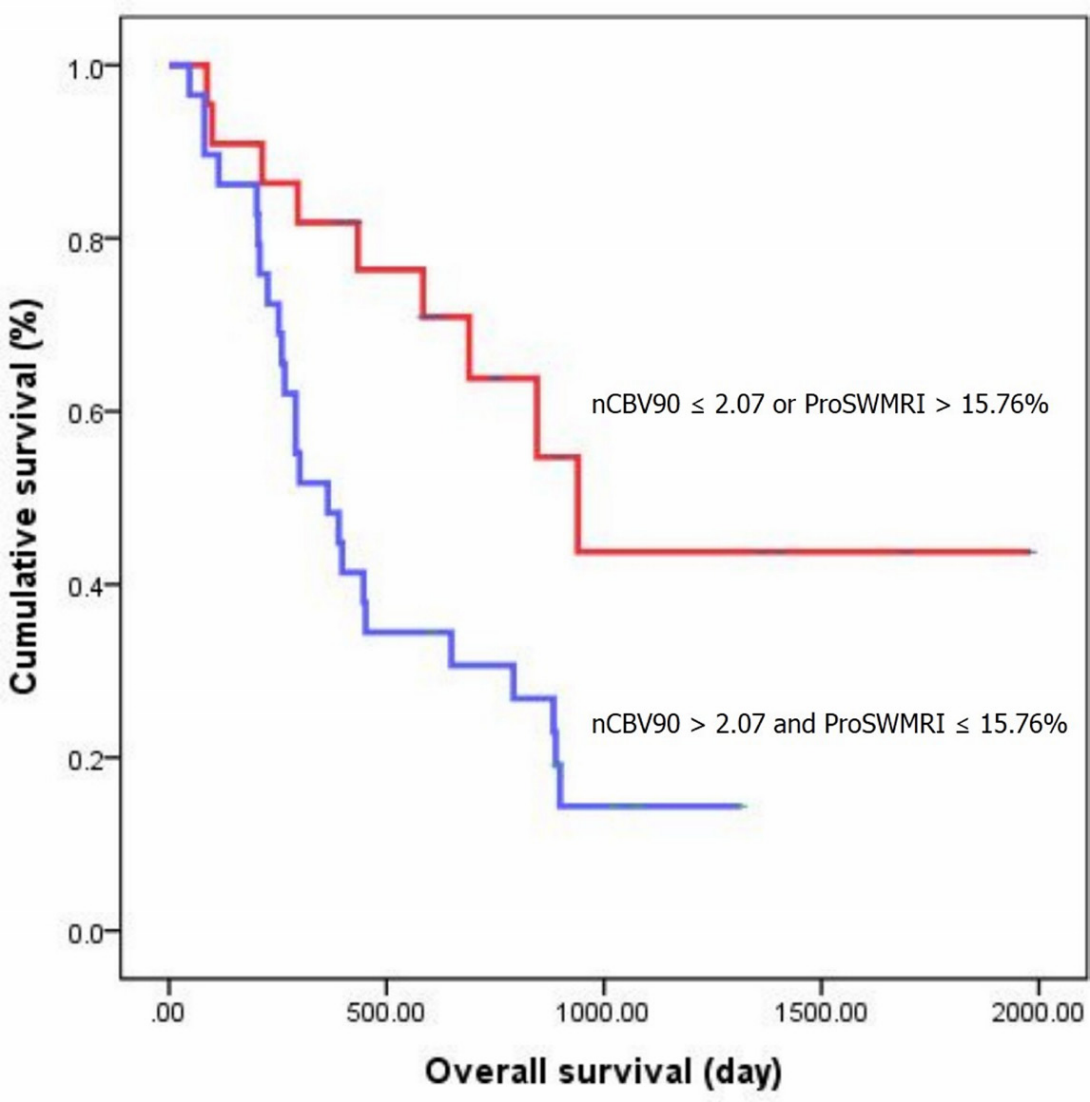
Kaplan-Meier curves between two groups of patients classified according to cutoff value of 2.07 for nCBV90 and of 15.76% for proSWMRI

The results of the Kaplan-Meier survival analysis with the log-rank test are shown in Figure 2. Significant differences were observed in the OS of the patient group that was dichotomized by the nCBV90 (cutoff value = 2.07) and proSWMRI (cutoff value = 15.76%). Median OS for patients with nCBV90 > 2.07 and proSWMRI ≤ 15.76% was 365 days (190, 539). OS at 6, 12 and 24 months for the subjects was 82.8, 62.1, 30.7 %, respectively. Median OS for patients with nCBV90 ≤ 2.07 or proSWMRI > 15.76% was 940 days (593, 1286). OS at 6, 12 and 24 months for the subjects was 90.9, 81.8, 63.8 %, respectively.

Table 3 summarizes the results of the histopathological quantitative assessment of the hemorrhagic foci within the contrast-enhancing portions of the MRI. More than 40 % of the hemorrhage foci were > 5 mm in diameter in the radionecrosis group, while 94 % of the hemorrhage foci were ≤ 5 mm in diameter in the recurrence group, which represents a significant difference (*P* < 0.001).

**Table 3:** Proportion of Hemorrhagic Foci Based on Size

	Radionecrosis							AVR (%)	Recurrence												AVR (%)	P value
**Patient No**.	1	2	3	4	5	6	7		1	2	3	4	5	6	7	8	9	10	11	12		
**ProSWMRI (%)**	28	69	76	79	80	88	88		0	0	0	01	1	5	7	7	7	13	17	31		
**Hemorrhagic foci ≤ 5 mm**, **%** **(No. of hemorrhage)**	91 (50)	46 (36)	53 (42)	40 (6)	71 (5)	45 (11)	52 (15)	57	100 (21)	100 (2)	100 (39)	100 (4)	100 (4)	100 (8)	91 (10)	88 (28)	79 (15)	79 (15)	92 (26)	95 (77)	94	0.001*
**Hemorrhagic foci > 5 mm**, **%** **(No. of hemorrhage)**	9 (4)	54 (45)	47 (37)	60 (9)	29 (2)	55 (13)	48 (14)	43	0 (0)	0 (0)	0 (0)	0 (0)	0 (0)	0 (0)	9 (1)	12 (6)	21 (8)	21 (8)	8 (5)	5 (6)	6	0.001*

Note: Data are the percentage and numbers in parentheses are the numbers of hemorrhage foci. Abbreviation: proSWMRI = proportion of dark signal intensity on susceptibility-weighted magnetic resonance imaging sequences

* The difference in the averages between the two groups was evaluated using the Mann-Whitney U test.

**Figure 3:**
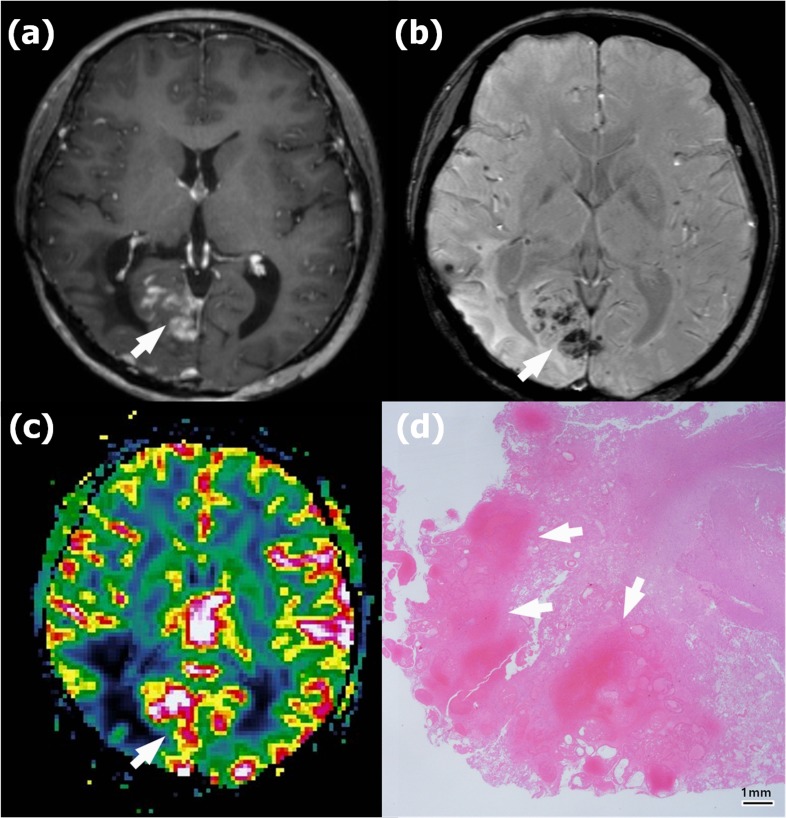
Recurrence in a 58-year-old woman with anaplastic astrocytoma in the left parietal lobe who underwent gross total resection and concomitant chemoradiotherapy (CCRT) with temozolomide **a**. Contrast-enhanced T1-weighted (CET1) magnetic resonance (MR) image obtained 14 months after CCRT completion shows a newly appearing enhancing lesion in the left parietal lobe (arrow). **b**. Susceptibility-weighted imaging demonstrates nearly no dark area in the corresponding lesion (arrow); the proportion of dark signal intensity was 0.30 %. **c**. The normalized relative cerebral blood volume map (nCBV) from dynamic susceptibility contrast perfusion-weighted imaging shows increased blood flow in the corresponding enhancing area (arrow) (calculated 90th percentile points in the cumulative nCBV histogram (nCBV90) = 5.13). **d**. Hematoxylin-eosin-stained histopathology (original magnification, X 10) shows multiple hemorrhages of ≤ 5 mm (arrows) within the recurrent anaplastic astrocytoma.

**Figure 4:**
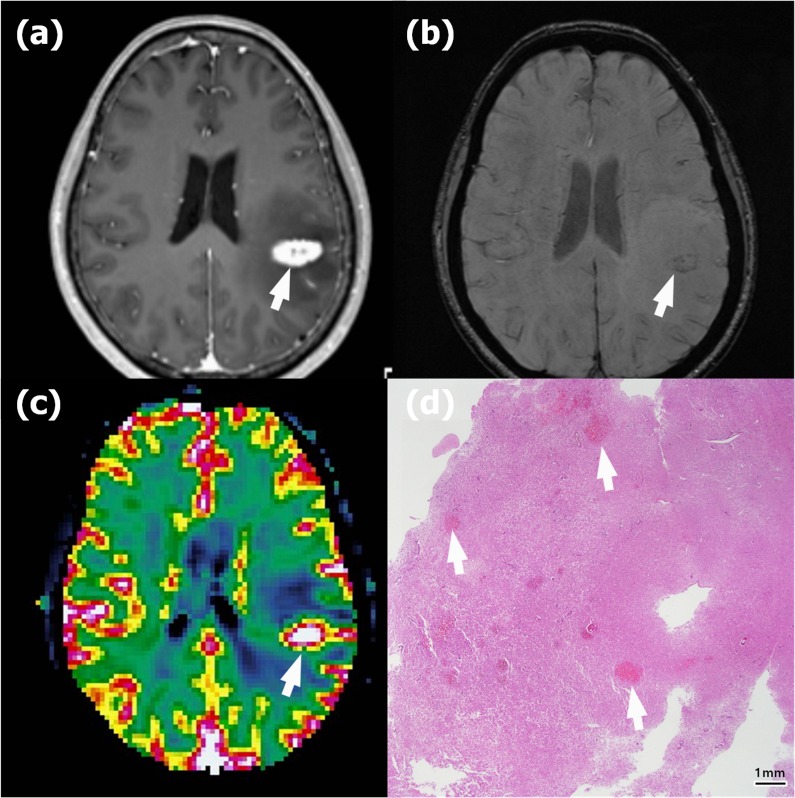
Radionecrosis in a 45-year-old woman with anaplastic astrocytoma in the right parietooccipital lobe who underwent gross total resection and concomitant chemoradiotherapy (CCRT) **a**. Contrast-enhanced T1-weighted (CET1) magnetic resonance (MR) image obtained 18 months after CCRT completion shows newly appearing multifocal enhancing lesions in the right occipital lobe (arrow). **b**. Susceptibility-weighted imaging demonstrates significant dark areas in the corresponding enhancing lesions (arrow); the proportion of dark signal intensity (proSWMRI) was 62.95 %. **c**. The normalized relative cerebral blood volume map (nCBV) from dynamic susceptibility contrast perfusion-weighted imaging shows increased blood flow in the corresponding enhancing area (arrow) (calculated 90th percentile points in the cumulative nCBV histogram (nCBV90) = 2.88). **d**. Hematoxylin-eosin-stained histopathology (original magnification, X 10) shows multiple hemorrhages of > 5 mm (arrows) within the radionecrosis.

**Figure 5:**
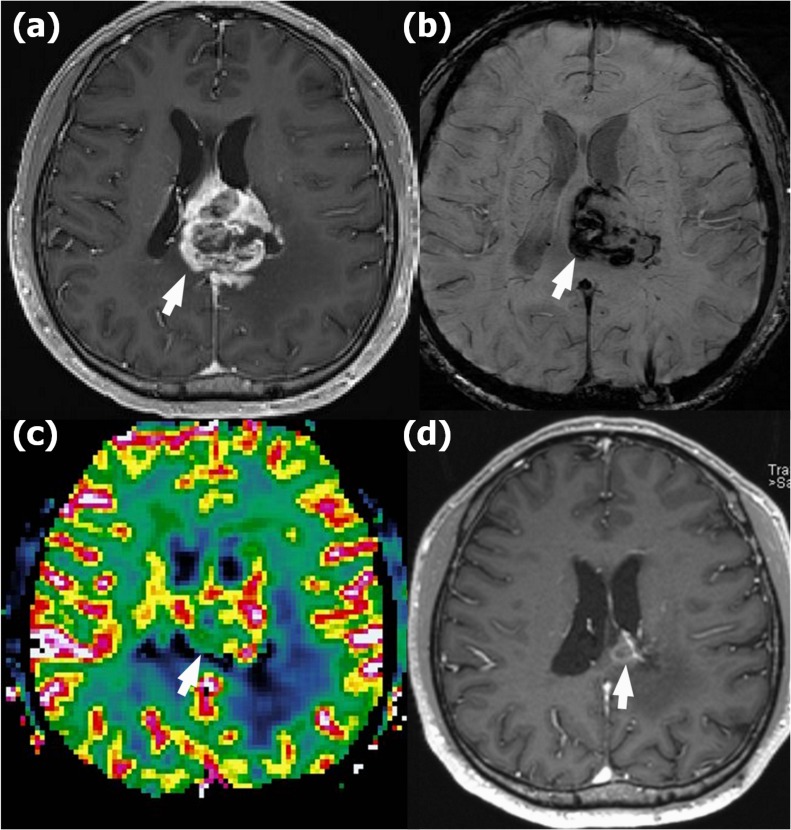
Radionecrosis in a 41-year-old man with glioblastoma in the left mid corpus callosum who underwent gross total resection and concomitant chemoradiotherapy (CCRT) **a**. Contrast-enhanced T1-weighted (CET1) magnetic resonance (MR) image obtained 18 months after CCRT completion shows newly appearing enhancing lesions in the left mid corpus callosum (arrow). **b**. Susceptibility-weighted imaging demonstrates significant dark areas in the corresponding enhancing lesions (arrow); the proportion of dark signal intensity (proSWMRI) was 40.5 %. **c**. The normalized relative cerebral blood volume map (nCBV) from dynamic susceptibility contrast perfusion-weighted imaging shows increased blood flow in the corresponding enhancing area (arrow) (calculated 90^th^ percentile points in the cumulative nCBV histogram (nCBV90) = 2.84). **d**. Follow-up CET1 MR image obtained 36 months after the first appearance of the enhancing lesion shows an interval decrease in the extent of the enhancing lesion (arrow), suggesting radionecrosis.

Representative images of recurrence and radionecrosis are shown in Figures 3, 4 and 5.

## DISCUSSION

In this study, we hypothesized that the combined use of SWMRI and DSC PWI could have the potential as prognostic factor for OS and improve the accuracy of the differential diagnosis of recurrence from radionecrosis in high-grade glioma patients. We found that the mean nCBV and nCBV90 were higher, and the proSWMRI was lower in the contrast-enhanced regions of recurrence compared with those of radionecrosis. Multivariate logistic regression analysis showed that only nCBV90 and proSWMRI were independent variables for this differentiation. The combination of nCBV90 and proSWMRI seems to have the potential to improve the accuracy of the differentiation of recurrence from radionecrosis compared with a single application of DSC PWI. Survival analysis showed that the combination of nCBV90 and proSWMRI can also be a prognostic factor for OS for high grade glioma patients

DSC PWI estimates tissue microvascular density by measuring rCBV [21]. In tumor recurrence, the blood volume increases because of neocapillary formation and dilatation of the existing vasculature [22]. Additionally, recent studies have supported this phenomenon that has been described as useful for distinguishing recurrence from RT-related changes. Hu et al. [12] proposed a threshold rCBV value of 0.71 for the optimized differentiation of tumor progression from RT-related changes with a sensitivity of 91.7 % and a specificity of 100 %. Young et al. reported similar results with a threshold rCBV value of 1.8 with a sensitivity of 100 % and a specificity of 75 % [23]; Prager et al. reported an optimized rCBV value of 1.74 with 91.9 % sensitivity and 66.7 % specificity [24]. Our results are consistent with the previous studies. The mean nCBV and nCBV90 values were significantly higher in recurrence than in radionecrosis (3.42 *vs*. 1.18, mean nCBV, *P* = 0.002; 5.98 *vs*. 1.97, nCBV90, *P* = 0.001, respectively). In the multivariate logistic regression analysis including the mean nCBV and nCBV90, only nCBV90 was considered as an independent variable for differentiation. This finding may suggest the potential advantage of the cumulative histogram method in analyzing high-grade glioma, which is intrinsically heterogeneous with various tumor grades in a single tumor [25]. Obtaining the total voxel values of a tumor would yield data that are objective in identifying the most aggressive portion of the tumor, providing better diagnostic accuracy than a comprehensive statistical value such as the mean value [26].

Radiation-induced hemorrhages are believed to be associated with radiation-induced vasculopathy [27, 28]. Nonoguchi et al [29] specifically reported that of 18 surgically resected radionecrosis specimens, telangiectasia was the most characteristic vasculature feature; microscopic bleeding was frequently observed. The histological examination in our study is consistent with the previous study, showing abundant hemorrhages in the radionecrosis lesions. In our study, most hemorrhages in recurrence were ≤ 5 mm in size, while more than 40 % of the hemorrhages in radionecrosis were > 5 mm in size. Our histological results suggest that a higher proportion of hemorrhages of > 5 mm in diameter may be responsible for the higher percentage of dark SI on SWMRI in radionecrosis.

SWMRI is a 3D gradient echo MR imaging technique that has proven to be more sensitive than conventional MRI in detecting hemorrhage [30]. Recent reports discussed the detection of radiation-induced hemorrhage with SWMRI as radiation-related changes [16, 17, 31, 32]. Zeng et al. [31] also described hypointense foci on SWI within the previously irradiated brain regions in glioma patients. To the best of our knowledge, the application of SWMRI for differentiating tumor recurrence from radionecrosis has not been reported in previous studies. We found that proSWMRI from the calculation of SWMRI was higher in radionecrosis than in recurrence (42.67 % *vs*. 9.3 %), suggesting a higher degree of hemorrhage in radionecrosis. In the ROC curve comparison, the combination of nCBV90 and proSWMRI achieved significantly higher specificity than that of nCBV90 alone (100.0 % (19/19) *vs*. 73.7 % (14/19), *P* = 0.046) while maintaining same accuracy (82.3% (42/51). This implicates adding proSWMRI would lower false positive rate ( = 1 - specificity) in differentiation recurrence from radionecrosis. In the subgroup analysis, among patients with nCBV90 > 2.07 (*n* = 33) who would have been regarded as recurrence with DSC PWI alone, the all radionecrosis group (*n* = 5) showed obvious hemorrhage (proSWMRI of > 32.9 %). The results of the ROC curve analysis and the subgroup analysis suggest that radionecrosis should be considered when new enhancing lesions combined with obvious hemorrhage appear on SWMRI after a long-term ( > six months) complete response in high-grade glioma patients, even with a high nCBV value.

Several studies previously reported that MRI parameters such as the ratio of T2/FLAIR to enhancing area [33] or PWI parameters can correlate with patients’ survival or response to the treatment [14, 34, 35]. The results of our study is in well line with previous studies and suggests that histogram analysis of nCBV maps and proportion of hemorrhage shown in SWMRI may be feasible for predicting overall survival in high grade gliomas patients.

We observed a higher incidence of glioblastoma (WHO grade IV) in the recurrence group than in the radionecrosis group, which likely reflects a more aggressive tendency of glioblastoma compared to other high-grade gliomas [1]. However, the multivariate logistic regression test results showed that MRI parameters such as nCBV and proSWMRI are more useful in determining whether a new enhancing lesion is tumor recurrence or radionecrosis, irrespective of the tumor grade.

Apart from the intrinsic limits of any retrospective study, our study had several other limitations. First, due to the small number of patients, the study's generalizability and statistical power are limited. However, we detected significant differences in DSC PWI and SWMRI between the two groups. Second, the evaluation of the non-enhancing infiltrative portion was limited. However, this study concentrated on a particular clinical setting—that is, a newly developed enhancing lesion mimicking recurrence. Therefore, only the nature of the enhancing lesion was considered. Third, quantitative susceptibility mapping (QSM) was not utilized in our study. QSM produces quantitative maps of tissue magnetic susceptibility using gradient recalled echo (GRE) phase data that can distinguish between blood products and calcium and quantify the extent of hemorrhage [36]. We believe that a future study using QSM would provide helpful information for the differentiation between recurrence and radionecrosis. Fourth, not all patients were histologically verified as recurrence or radionecrosis. However, due to the rapid growth of the high grade gliomas, progressive enlargement of the initial enhancing lesion within a 6-month follow-up period was considered sufficient to reach a safe estimation on the diagnosis of recurrence . Lastly, two different susceptibility-weighted MR sequences were included in our study (SWI, Siemens,; SWAN, General Electric;). Each of two sequences presents a different technical background and differences in terms of contrast within the image which may result in heterogeneous sensitivity in detecting micro hemorrhages [37].

In conclusion, this study revealed that histogram analysis based on the nCBV of the entire newly enhancing lesion may be a better diagnostic tool than the mean nCBV in differentiating recurrence from radionecrosis; the combination of DSC PWI and SWMRI seems to have the potential to be a prognostic factor for OS and to lower false positive rate in differential diagnosis in high-grade glioma patients who develop new enhancing lesions after a long-term complete response.

## MATERIALS AND METHODS

This retrospective study was approved by the institutional review board of our institution; informed consent was waived.

### Patient selection

We selected from our radiology report database 233 patients who previously underwent brain RT for high-grade glioma and who had undergone serial follow-up 3T brain MRI in our institution between January 2008 and April 2015. The inclusion criteria were as follows: (a) a histopathologic diagnosis of high-grade glioma according to the World Health Organization criteria; (b) the patient underwent RT or gamma knife surgery followed by near-total tumor removal of the brain tumor; (c) follow-up MR imaging at 3T was performed with contrast enhancement and included DSC PWI and SWMRI; (d) follow-up MRI showed newly developed enhancing lesions inside the radiation field after intravenous injection of gadolinium-based contrast media; and (e) the post-irradiation period was longer than six months.

We excluded 182 patients for the following reasons: (a) inadequate MR image quality (*n* = 44); (b) no newly appearing lesions on the follow-up MR images (*n* = 67); (c) newly visible enhancing lesions did not meet the criteria for measurable disease as defined according to the RANO criteria (dimensionally contrast-enhancing lesions with clearly defined margins by MRI scans, with two perpendicular diameters of at least 10 mm) (16) (*n* = 27); (d) newly developed lesions occurring less than six months after the completion of RT (*n* = 11); and (e) loss to follow-up (*n* = 33).

Ultimately, 51 patients (30 men and 21 women; mean age, 52.9 years; age range, 25-72 years) who were diagnosed with glioblastoma (*n* = 29; isocitrate dehydrogenase (IDH) -wildtype (*n* = 7), IDH-mutant (*n* = 7), Not otherwise specified (NOS) (*n* = 15)), anaplastic astrocytoma (*n* = 15; IDH-wildtype (*n* = 1), IDH-mutant (*n* = 2), NOS (*n* = 12)), anaplastic oligodendrogliomas (*n* = 3, NOS), and anaplastic oligoastrocytoma (*n* = 4, NOS) were included . Thirty-two patients were identified as having a recurrence, while 19 patients were diagnosed with radionecrosis by either radiologic determination or histologic confirmation. Four patients underwent gamma knife surgery (recurrence (*n* = 1), radionecrosis (*n* = 3)) . In addition, clinical outcomes including patients’ current status (dead, alive, or follow-up loss) and survival days after the initial appearance of measurable enhancement on follow up MRI were collected. Overall follow-up survival data was completed by reviewing electronic medical record of our hospital as well as by contacting the Resident Service Division of the Ministry of Public Administration and Security. The endpoints of this study were either the patient's death or May 31, 2016. For methylguanine methyltransferase (MGMT), a DNA repair enzyme that removes alkyl groups from guanine residues, the promoter methylation status was investigated by using the methylation-specific polymerase chain reaction technique.

### Image acquisition

Follow-up MRI studies of all patients were performed using one of two 3 T MR imaging scanners (*n* = 25 [recurrence = 18 and radionecrosis = 7]; Signa Excite; GE Medical Systems, Milwaukee, WI, USA; and *n* = 26 [recurrence = 14 and radionecrosis = 12]; Verio; Siemens Medical Solutions, Erlangen, Germany) with an eight-channel head coil. SWMRI included either SWI or SWAN in the current study . The imaging protocol included spin-echo (SE) T1-weighted images (T1WI), fast SE (FSE) T2-weighted images (T2WI), fluid-attenuated inversion recovery (FLAIR) images, SWI or SWAN, DSC PWI with gadobutrol (Gadovist, Bayer Schering Pharma, Berlin, Germany), and subsequent contrast-enhanced (CE) SE T1WI. The MRI parameters were as follows: 558-650/8-20 ms/70-90°/384 × 192-212 (TR/TE/FA/matrix) for SE T1WI; 4500-5160/91-106.3 ms/90-130°/448-640 × 220 for FSE T2WI; 9000-9900/97-162.9 ms/90-130°/199-220 × 220 for FLAIR images; 28/20 ms/15°/448 × 255 for SWI; and 78.8/49.8 ms/15°/240 × 240 for SWAN. The other parameters were as follows: section thickness, 5 mm with a 1 mm gap and field of view (FOV), 240 × 240 mm. DSC PWI was performed with a single-shot gradient-echo echo-planar imaging sequence during the intravenous injection of the contrast agent. The imaging parameters of the DSC PWI were as follows: TR/TE, 1500/30-40 ms; FA, 35-90°; FOV, 240 × 240 mm; 140-20 sections; matrix, 128 × 128; section thickness, 5 mm intersection gap, 1 mm; and voxel resolution of 1.86 × 1.86 × 5 mm. For each section, 60 images were obtained at intervals equal to the repetition time. After four to five time points, a bolus of gadobutrol, at a dose of 0.1 mmol/kg of body weight and a rate of 4 mL/sec, was injected with an MR-compatible power injector (Spectris; Medrad, Pittsburgh, PA, USA). The contrast material bolus was followed by a 30 mL bolus of saline, which was administered at the same injection rate.

### Determination of lesions

Radiologic determination or histologic confirmation was performed to determine whether the lesions represented recurrence or radionecrosis. The radiologic determination was made by two neuroradiologists (J.H.K. and T.J.Y., with 13 and 10 years of brain MRI experience, respectively) who independently reviewed each patient's MR images. The diagnosis of recurrence was established if a progressive increase in the contrast-enhancing lesions was seen on the second and third follow-up MR studies (with an interval of 3 months) after the initial progress seen on the first follow-up MR study . The diagnosis of radionecrosis was made if a decrease or stabilization of the contrast-enhancing lesions for a minimum of 6 months was observed on the subsequent follow-up MR studies. For MRI in which the two radiologists’ findings were discrepant, a consensus was reached. In patients who underwent reoperation or stereotactic biopsy for the new enhancing lesions, histologic confirmation was available. The final determination for the diagnosis of recurrent GBM or radiation necrosis was decided on the basis of the following criteria [38]: (a) Samples containing a mixture of both recurrent GBM and radiation necrosis were classified as showing recurrent GBM, regardless of the degree of admixture; (b) only samples with pure radiation change (in the absence of tumor criteria) were categorized as showing radiation necrosis; and (c) the presence of a few isolated, scattered atypical cells did not qualify for tumor categorization if other neoplastic features were absent.

### Quantitative image analysis

The MR data for the DSC PWI and SWMRI were digitally transferred from the picture archiving and communication system workstation to a personal computer for further analyses. Relative CBVs (rCBVs) were determined using a dedicated software package (NordicICE; NordicImagingLab, Bergen, Norway) with an established tracer kinetic model applied to the first-pass data [21, 39]. First, to minimize patient motion during the dynamic scans, realignment was performed. Second, the gamma-variate function, which is an approximation of the first-pass response as it would appear in the absence of recirculation, was used to fit the 1/T2* curves to reduce the effects of recirculation. To reduce the effect of contrast agent leakage, the dynamic curves were mathematically corrected [40]. After the correction of recirculation and contrast agent leakage, the rCBV was extracted using numeric integration of the curve. To minimize variances in the rCBV value in each patient, the pixel-based rCBV maps were normalized. Every rCBV value in a specific section was divided by the rCBV value in the normal white matter, contralateral to the enhancing lesion, as selected by a neuroradiologist (S.H.C.) [41].

The quantitative image analysis was independently performed by two radiologists (S.H.C., T.H.K.). The regions of interest (ROIs) were selected by connecting dotted lines with the software that contained the entire enhancing lesion of the contrast-enhanced T1WI on every continuous section of the co-registered images. Small or thin-rim enhancing lesions that did not fulfill the RANO criteria for measurable disease were excluded (16). Any areas of small vessels and necrosis were also carefully excluded from the ROIs. Then co-registrations of the CE T1W images between the normalized CBV (nCBV) maps and between the SWMRI were performed based on geometric information stored in the respective data sets using the dedicated software (NordicICE). The differences in slice thickness between images were adjusted automatically with the re-slicing and co-registration method. The CE T1W images of the nCBV maps were displayed as color overlays, and those of the SWMRI maps were displayed as gray scale overlays. Next, histogram analysis was performed for the nCBV values. First, the nCBV histograms were plotted with nCBV on the x-axis, with a bin size of 0.1; the y-axis was expressed as a percentage of the total lesion volume by dividing the frequency in each bin by the total number of analyzed voxels. Second, for further quantitative analysis, cumulative nCBV histograms were obtained from the nCBV histograms, in which the cumulative number of observations in all of the bins up to the specified bin was mapped on the y-axis as percentages. In the cumulative nCBV histograms, the 70th, 90th, 95th and 99th percentile points (nCBV70, nCBV90, nCBV95 and nCBV99, respectively) were derived (the Xth percentile point is the point at which X % of the voxel values that form the histogram are found to the left of the histogram) [42]. The nCBV70 was arbitrary chosen as one of a cutoff value to reflect high grade gliomas with less marked degree of hyperperfusion or to exclude disproportionate contribution by vessels at the extreme top of the histogram curve [43]. Each percentile point was compared using areas under the receiver operating characteristic (ROC) curves at the points. Using this process, we chose the Xth percentile point of the cumulative histogram that showed the highest value of the areas under the ROC curve.

**Figure 6:**
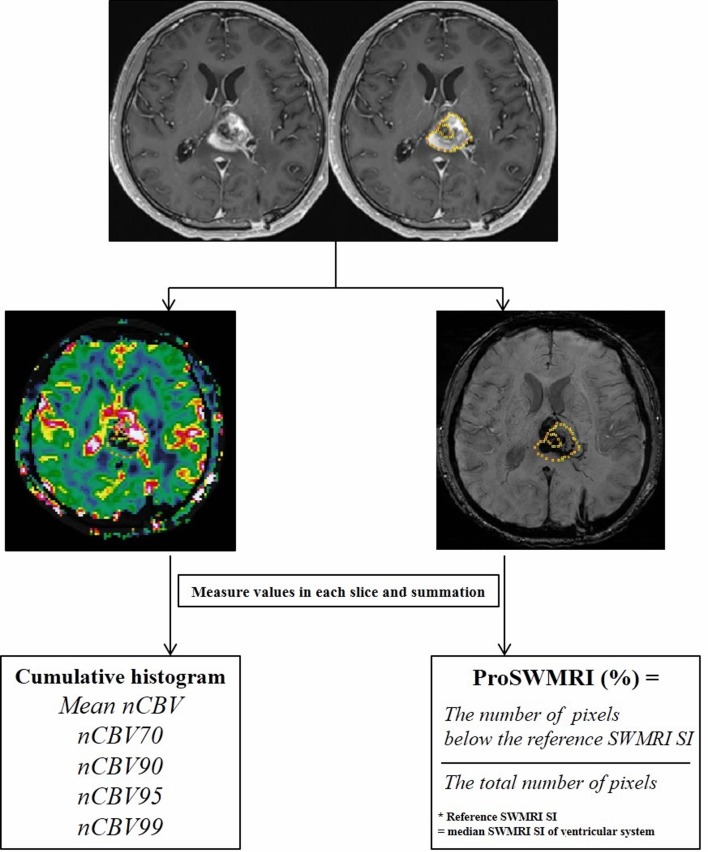
Flow chart of quantitative image analysis Region of interest (ROI) was manually selected in each section of the enhancing lesions and was semi-automatically co-registered with the normalized relative cerebral blood volume map (nCBV) and susceptibility-weighted magnetic resonance imaging sequences (SWMRI) map. The volume of interest was determined by the summation of each slice; nCBV values and SWMRI values for the entire enhancing lesion were obtained. Cumulative histogram analysis was performed for whole nCBV values. In the cumulative nCBV histograms, mean nCBV, and the 70th, 90th, 95th and 99th percentile points (nCBV70, nCBV90, nCBV95 and nCBV99, respectively) were derived. For SWMRI values, by setting the median SWMRI SI of the ventricular system as the reference, the proportion of SWMRI (proSWMRI) was calculated by dividing the number of pixels below the reference SWMRI SI by the total number of pixels. Note: ROI = region of interest, SWMRI = susceptibility-weighted magnetic resonance imaging sequences, SI = signal intensity, CBV = cerebral blood volume, the Xth percentile point = point at which X% of the voxel values that form the histogram are found to the left of the histogram.

For SWMRI, every pixel value was extracted from each ROI drawn around the entire contrast-enhanced region for each transaxial section. Among four different sets of images generated by Siemens scanner, we utilized the processed SWMRI magnitude image created by combining the phase and the magnitude map [15]. To define the reference signal intensity (SI), the ROI was placed in the ventricular system of the SWMRI map where the median SI was calculated. The proportion of dark SI of the lesions on SW images (proSWMRI) was defined as the percentage of the pixels with values below the reference SI (100 x the number of the pixel with values below the reference SI / the total number of the extracted pixel) [44]. A summary flow chart of the quantitative image analysis is described in Figure 6.

### Histopathologic correlation

All available specimens from the 19 patients (12 of 32 recurrences, 7 of 19 radionecrosis) were histologically examined by one pathologist (J.K.W.) for the quantification of hemorrhage. For this quantification, we included all sections stained with hematoxylin and eosin covering the whole contrast-enhancing portions on MRI in each patient. Hemorrhagic foci within the sections were categorized according to the following size criteria in the longest diameter; ≤ 5 mm and > 5 mm. Then, the number of hemorrhagic foci as well as proportion of the number of hemorrhage in each size was recorded.

### Statistical analysis

All statistical analyses were performed using MedCalc software (v 15.8.0; MedCalc Software, Mariakerke, Belgium) and SPSS software (v 21.0 for Windows, SPSS, Chicago, Ill). The results with a P value of less than .05 were considered statistically significant.

The clinical characteristics were compared between the recurrence and radionecrosis groups using Fisher's exact test for categorical variables and unpaired Student's *t*-test for non-categorical data..

Unpaired Student's *t*-test was used to compare the mean nCBV, histogram parameters of nCBV and proSWMRI of the recurrence and radionecrosis. Inter-observer agreement on the quantitative analysis was assessed by the interclass correlation coefficient and the coefficient of variation . To assess the most promising Xth percentile point of the cumulative histogram, the areas under the ROC curves (AUCs) with histogram parameters of nCBV were compared using the method of DeLong et al (22). ROC analysis was performed to determine the best cutoff values for the mean nCBV, histogram parameters and proSWMRI that proved to be substantial predictors in differentiating recurrence from radionecrosis. Next, a stepwise multivaraible logistic regression model was applied to determine the best predictors of the differential diagnosis between the recurrence and radionecrosis. With these data, we determined the diagnostic performance of the combination of the best predictors for the differentiation. Fisher's exact test was performed to compare sensitivities and specificities of the predictors. The leave-one-out cross-validation (LOOCV) test was also performed to evaluate the accuracy of the combination of the best predictors.

Kaplan-Meier survival analysis and the log-rank test for group comparison were performed regarding the best cutoff of the histogram parameters that showed a difference between two groups in the ROC analysis to provide median overall survival (OS) estimates and time specific rates.

The proportion of hemorrhage with size of ≤ 5 mm or > 5 mm in the longest diameter was compared between recurrence and radionecrosis by using Mann-Whitney U test.
